# Effect of stilbene derivative on superoxide generation and enzyme release from human neutrophils *in vitro*

**DOI:** 10.2478/v10102-012-0012-7

**Published:** 2012-06

**Authors:** Tatiana Mačičková, Jana Pečivová, Juraj Harmatha, Klára Sviteková, Radomír Nosáľ

**Affiliations:** 1 Institute of Experimental Pharmacology & Toxicology, Slovak Academy of Sciences, SK-84104 Bratislava, Slovakia; 2 Institute of Organic Chemistry and Biochemistry, AS CR, CZ-16610 Praha, Czech Republic; 3 National Transfusion Service, SK-83101Bratislava, Slovak Republic

**Keywords:** pterostilbene, human neutrophils, superoxide generation, myeloperoxidase

## Abstract

Neutrophils represent the body′s primary line of defense against invading pathogens. They most rapidly reach the site of injury or infection, liberate antimicrobial proteins, proteases and produce reactive oxygen species. Prolonged or excessive liberation of these very effective and toxic substances could intensify the inflammatory process and enhance tissue damage in many diseases, such as allergies, infections and rheumatoid arthritis. Pterostilbene belongs to stilbenoids, structural analogues of resveratrol, which act as natural protective agents in defending the plant against viral and microbial attack. It possesses anticancerous, antidiabetic and anti-inflammatory properties.

The study provides new information on the effect of pterostilbene [0.01–100 µmol/l] on superoxide generation in and myeloperoxidase (MPO) release from azurophil granules of isolated human neutrophils. PMA [1µmol/l], which activates NADPH-oxidase via protein kinase C, was used for stimulation of neutrophils Unstimulated cells showed neither superoxide generation nor myelopereoxidase release after preincubation with the drug studied. Pterostilbene dose dependently decreased superoxide generation in and MPO release from stimulated human neutrophils, however a significant decrease was recorded only in the concentration 100 µmol/l. The effect of pterostilbene was more pronounced on superoxide generation in comparison to MPO release. Our results suggest that the effect of pterostilbene may prove beneficial in controlling inflammation.

## Introduction

During inflammation, professional phagocytes, *e.g.* neutrophils, are recruited from the circulation to tissues where they produce reactive oxygen species (ROS) using the membrane-associated enzyme NADPH-oxidase. In the process of oxidative burst, neutrophils produce a variety of cytotoxic products, *e.g.* superoxide, hydrogen peroxide and hypochloric acid (El-Benna *et al.*, 2008). Together with proteolytic enzymes released from activated neutrophils in the process of degranulation, ROS have been recognized as an important factor contributing to neutrophil-mediated injury (Babior, 2000; Lastra & Villegas, 2005).

**Figure 1:**
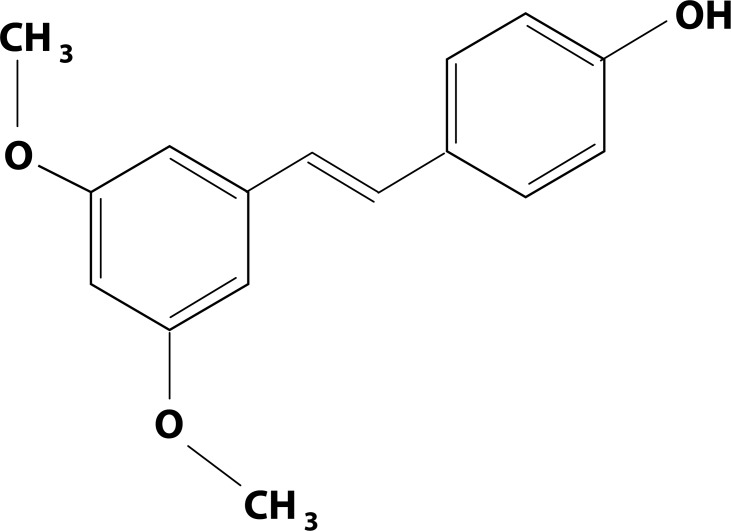
Structure of pterostilbene.

Pterostilbene, trans-3,5-dimethoxy-4′-hydroxy stilbene (Figure 1), is a natural substance from the stilbenoid group, widespread in a variety of plants, in leaves and grapes of Vitis vinifera, berries of Vaccinium spp., Pterocarpus marsupianum, etc. (Paul *et al.*, 1999; Grover *et al.*, 2005). It is chemically related to resveratrol, which is well known for its antioxidative activity. Structural analogues of resveratrol possess some of the beneficial effects of the parent drug and may provide even further benefits.

Pterostilbene was found to be an effective anti-proliferative agent against lung cancer cell lines and its anticancer properties were demonstrated in melanoma, breast, gastric and hepatocellular carcinomas (Remsberg *et al.*, 2008; Schneider *et al.*, 2010). Chronic treatment with pterostilbene remarkably reduced pathological changes observed in the liver and kidney of diabetic rats, confirming the antioxidant properties to pterostilbene (Satheesh & Pari, 2006). Pterostilbene inhibited the production of hydroxyl radicals but was ineffective against peroxyl radicals on using ORAC/HORAC assays in cell free system (Perečko *et al.*, 2010).

The plasmatic level of the immunological marker MCP-1 formed in rats with adjuvant arthritis was decreased by pterostilbene (Mihalová *et al.*, 2010). Pterostilbene exerted a partial effect on luminol-enhanced chemiluminescence of the joint of rats with adjuvant arthritis in comparison with untreated animals (Mačičková *et al.*, 2010). It significantly decreased extra- and intra-cellular chemiluminescence of isolated human neutrophils and oxidative burst in whole human blood (Perečko *et al.*, 2008). In the model of arthritic rats, pterostilbene significantly reduced the number of neutrophils, though the oxidant concentration in blood was only slightly decreased (Perečko *et al.*, 2010, Perečko *et al.*, 2010a).

In this paper we investigated the effect of pterostilbene on superoxide generation in and MPO release from azurophil granules of human neutrophils after PMA activation.

## Material and methods

Pterostilbene was prepared by targeted regioselective synthesis, purely as trans isomer (Šmidrkal *et al.*, 2010) in the Institute of Organic Chemistry and Biochemistry, Prague, Czech Republic. Dextran T500 was purchased from Pharmacia Fine Chemicals, Lymphoprep from Nycomed Pharma AS, cytochalasin B from Merck. All other chemicals used were from Sigma-Aldrich.

### Isolation of human neutrophils

Blood was collected by venipuncture from healthy male volunteers and put into vials with 3.8% trisodium citrate. Erythrocytes were removed by dextran sedimentation and centrifugation on Lymphoprep by the modified Boyum′s method (Drábiková *et al.*, 2002). The final suspension contained more than 96% viable cells, as evaluated by trypan blue exclusion. The number of neutrophils for superoxide and MPO determination was 1x10^6^ and 2x10^6^/sample, respectively.

### Superoxide determination

Superoxide dismutase inhibitable reduction of cytochrome c was used to measure superoxide generation in isolated human neutrophils. The superoxide production was calculated as described by Pečivová *et al.* (2007). Superoxide generation was determined by means of microplate spectrophotometry at 550 nm.

### Myeloperoxidase release

Myeloperoxidase release was assayed by determining the oxidation of o-dianisidine in the presence of hydrogen peroxide in a microplate spectrophotometer at 463 nm (Pečivová *et al.*, 2006).

### Statistical analysis

All values are given as means of 6–8 experiments ± SEM. Statistical significance of differences between means was established by Student′s t-test and *p*-values below 0.05 were considered statistically significant.

## Results

After nonreceptor PMA [1µmol/l] stimulation the effect of pterostilbene was studied on human neutrophil functions – respiratory burst and degranulation. Respiratory burst was investigated during testing the effect of pterostilbene on superoxide – the first step of ROS generation, and on degranulation as MPO release from azurophil granules of human neutrophils. On unstimulated isolated human neutrophils, preincubation with pterostilbene [0.01–100µmol/l] had no effect on superoxide generation and MPO release.

**Figure 2:**
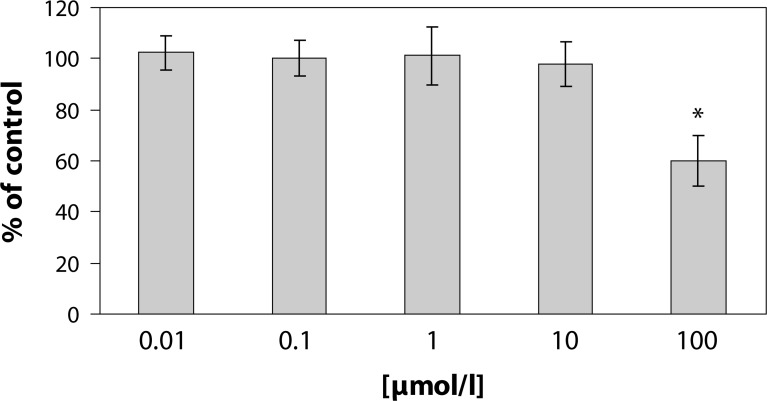
Effect of pterostilbene on PMA [1 µmol/l, 15 min/37°C] stimulated superoxide generation in human neutrophils. Results are mean ± SEM, n = 6–8, *p<*0.05 compared to control value without pterostilbene. Results are expressed as percentage of absolute average control value (PMA) of superoxide generation 50.41±2.14nmoles/10^6^ neutrophils /min.

**Figure 3:**
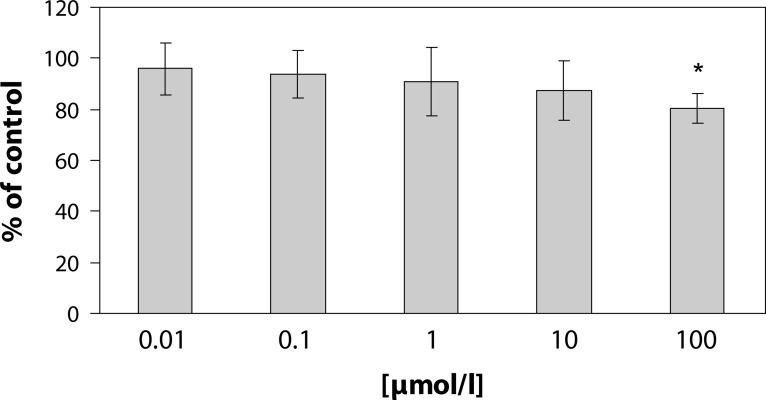
Effect of pterostilbene on PMA [1 µmol/l, 15 min/37°C] stimulated myeloperoxidase release from human neutrophils. Results are mean ± SEM, n = 6–8, *p<0.05 compared to control value without pterostilbene. Results are expressed as percentage of absolute average control value (PMA) of MPO release 3.09 ± 0.29 ΔA/Δt (calculated as area under curve).

Figure 2 shows the effect of pterostilbene on superoxide generation in PMA stimulated human neutrophils. Pterostilbene dose-dependently decreased superoxide generation, however a significant decrease was recorded with 100µmol/l pterostilbene only. Incubation of neutrophils with pterostilbene revealed good concentration dependence, yet significant decrease of MPO release was observed in the highest concentration [100 µmol/l] only (Figure 3). The effect of pterostilbene was more pronounced on superoxide generation in comparison to MPO release.

## Discussion

Neutrophils and macrophages represent professional phagocytic cells. When appropriately stimulated, they undergo dramatic physiological and biochemical changes resulting in phagocytosis, chemotaxis and degranulation with the activation of ROS production known as respiratory burst (Lastra &Villegas, 2005).

The antioxidant activity of resveratrol and its analogues depends significantly on the position of the hydroxyl groups. Structure-activity relationship analysis indicated that structural determinants are necessary for the antioxidant activity of resveratrol and its analogues, *i.e.* the hydroxyl group required in the 4′position, as well as a trans-configuration of the ethylene functional group in the stilbene skeleton (Stivala *et al.*, 2001; Ovesná & Horváthová, 2005; Cheng *et al.*, 2006; Hasiah *et al.*, 2011).

Previous investigations have found that pterostilbene has antioxidant, anti-inflammatory and anticancer properties similar to those of resveratrol (Rimando *et al.*, 2002; Remsberg *et al.*, 2008). Melanoma, breast, gastric and hepatocellular carcinomas were found to be pterostilbene sensitive malignancies (Remsberg *et al.*, 2008). Pterostilbene is an effective anti-proliferative agent against lung cancer cell lines (Schneider *et al.*, 2010) and colon cancer (Rimando & Suh, 2008). Comparably to resveratrol, it was reported to have antiproliferative and cytostatic activities on different cell lines. Moreover, it did not exhibit cytotoxic activity in the concentrations tested (Stivala *et al.*, 2001), as corroborated by Perečko *et al.* (2008) using the ATP-cytotoxicity test.

Pterostilbene may be a potent chemoprotective agent and dietary exposure to pterostilbene has been assumed to reduce harmful effects of oxidative stress (Kim *et al.*, 2009). Satheesh & Pari (2006) reported that chronic treatment with pterostilbene remarkably reduced pathological changes observed in the liver and kidney of diabetic rats, confirming the antioxidant properties of pterostilbene.

Over recent years, the antioxidant properties of pterostilbene have attracted the interest of many research groups, however as far as we know, the effect of pterostilbene on superoxide generation in and MPO release from human neutrophils has not yet been investigated. The goal of this study was to examine the effect of pterostilbene on human neutrophil functions – respiratory burst and degranulation after nonreceptor PMA stimulation. PMA, areceptor bypassing stimulus, activates NADPH-oxidase in neutrophils *via* protein kinase C (Klink *et al.*, 2009). Our aim was to assess the effect of pterostilbene on superoxide generation in human neutrophils and on degranulation as MPO release. Superoxide radicals are the first step of ROS generation and precursors of other ROS. MPO is a major constituent of the azurophilic granules of neutrophils. It is the most abundant enzyme in neutrophils and a major NO scavenger and marker of oxidative stress (Brennan & Hazen, 2003). Along with NADPH-oxidase, MPO is involved in the formation of ROS and oxidation of biological material.

In our experimental conditions, pterostilbene dose-dependently decreased superoxide generation, however a significant decrease was recorded only with pterostilbene concentration of 100 µmol/l. Our present results showed that incubation of neutrophils with pterostilbene caused significant inhibition of MPO release from stimulated human neutrophils induced by PMA in the concentration of 100 µmol/l. In comparison to MPO release, the effect of pterostilbene was more pronounced on superoxide generation.

Pterostilbene, a rather lipophilic analogue of resveratrol, was shown to be as effective as resveratrol in the assay of peroxyl radicals scavenging activity. It reduced singlet-oxygen-induced peroxidation and protected the normal human fibroblast and erythrocyte membranes against lipid peroxidation (Stivala *et al.*, 2001; Rimando *et al.*, 2002; Mikstacka *et al.*, 2010).

Remsberg *et al.* (2008) demonstrated the ability of pterostilbene to scavenge 1,1-diphenyl-2-picrylhydrazyl (DPPH) free radicals and to decrease blood glucose. It also inhibited production of hydroxyl radicals but was ineffective against peroxyl radicals using HORAC/ORAC assays in cell free system (Perečko *et al.*, 2010).

Stilbenes have been shown to inhibit the activity of ROS. *In vitro* studies, comparing pterostilbene, resveratrol and pinosylvin, showed that pterostilbene inhibited PMA stimulated whole human blood chemiluminescence in dose dependent manner. Significant inhibition was reached at the concentration of 1 µmol. The effect of pterostilbene on extra- and intra-cellular chemiluminescence in PMA activated human neutrophils targeted mainly extracellular ROS, which are responsible for tissue damage during chronic inflammation. Despite the highest lipophilicity among the substances tested, pterostilbene was the least effective against intracellular chemiluminescence of isolated human neutrophils (Perečko *et al.*, 2008). Pterostilbene inhibited extra- and intra-cellular production of cytotoxic ROS in neutrophils wihout affecting of their viability (*Perečko et al.,* 2009). In the attempt to elucidate the molecular mechanism of pterostilbene Perečko *et. al.* ( 2010) examined its effect on activation/phosphorylation of protein kinase C, an activation marker and antecedent step of phosphorylation of NADPH-oxidase subunits. Pterostilbene did not change the phosphorylation of protein kinase C a /ß II of isolated human neutrophils, suggesting involvement of other mechanisms of ROS generation reduction, *e.g.* inhibition of NADPH-oxidase.

In our previous study, we tested the efficacy of pterostilbene in reducing the oxidative stress caused by auto-immune inflammatory processes in the rat adjuvant arthritis model. Pterostilbene exerted only a partial effect on luminol-enhanced chemiluminescence of the joint and had no effect on MPO activity in hind paw joint homogenates of rats with adjuvant arthritis in comparison with untreated animals (Mačičková *et al.*, 2010). The better direct effect of pterostilbene in the concentration of 0.01–100 µmol/l on MPO release from isolated human neutrophils in comparison to the effect of pterostilbene on MPO activity in the rat model of adjuvant arthritis (Mačičková *et al.*, 2010) may be caused by interspecies distinctions and by different actual concentrations of pterostilbene in the tissues. Pterostilbene slightly decreased oxidant concentration in blood of arthritic rats and it significantly reduced the number of neutrophils (Perečko *et al.*, 2010, Perečko *et al.*, 2010a). Pterostilbene suppressed lipopolysaccharide-induced up-expression of iNOS and COX-2 in murine macrophages (Pan *et al.*, 2008).

In agreement with our results, the natural compound pterostilbene was identified as effective in reducing the oxidative burst of human neutrophils during their activation *in vitro*. This effect does probably not include the protein kinase C phosphorylation pathway (Perečko *et al.*, 2008; 2010).

We conclude that the effect of pterostilbene on superoxide generation in and MPO release from PMA activated human neutrophils appears to be a rather complex process. Partially, pterostilbene may reduce the activity of enzymes producing ROS (NADHP-oxidase; MPO) yet it also interferes with ROS already generated. In this way, pterostilbene may increase the protection of host tissues and prove beneficial in controlling inflammation.
